# Quantifying Catch Rates, Shark Abundance and Depredation Rate at a Spearfishing Competition on the Great Barrier Reef, Australia

**DOI:** 10.3390/biology11101524

**Published:** 2022-10-18

**Authors:** Adam Smith, Al Songcuan, Jonathan Mitchell, Max Haste, Zachary Schmidt, Glenn Sands, Marcus Lincoln Smith

**Affiliations:** 1 Reef Ecologic, Townsville, QLD 4810, Australia; 2 Department of Agriculture and Fisheries, Queensland Government, Dutton Park, QLD 4102, Australia; 3 Townsville Skindiving Club, South Townsville, QLD 4810, Australia; 4 Department of Biological Sciences, Faculty of Science and Engineering, Macquarie University, Macquarie Park, NSW 2019, Australia

**Keywords:** spearfishing, pelagic, fish, shark, depredation, sustainability citizen science

## Abstract

**Simple Summary:**

Catch-per-unit-effort at a spearfishing competition in North Queensland, Australia was 1.25 fish per hour. Citizen scientists reported 358 sharks comprising of 10 species/groups, with 3.11 sharks observed per hour. Twelve percent of all sharks sighted in this study were regarded as potentially dangerous to humans. This is the first scientific study of shark depredation for spearfishing. *C. amblyrhynchos* were primarily responsible for depredation, with 5.9% of speared fish taken. Sixty percent of interviewees perceived that shark numbers have increased in the past 10 years.

**Abstract:**

We developed and applied a method to quantify spearfisher effort and catch, shark interactions and shark depredation in a boat-based recreational spearfishing competition in the Great Barrier Reef Marine Park in Queensland. Survey questions were designed to collect targeted quantitative data whilst minimising the survey burden of spearfishers. We provide the first known scientific study of shark depredation during a recreational spearfishing competition and the first scientific study of shark depredation in the Great Barrier Reef region. During the two-day spearfishing competition, nine vessels with a total of 33 spearfishers reported a catch of 144 fish for 115 h of effort (1.25 fish per hour). A subset of the catch comprised nine eligible species under competition rules, of which 47 pelagic fish were weighed. The largest fish captured was a 34.4 kg Sailfish (*Istiophorus platypterus*). The most common species captured and weighed was Spanish Mackerel (*Scomberomorus commerson*). The total weight of eligible fish was 332 kg and the average weight of each fish was 7.1 kg. During the two-day event, spearfishers functioned as citizen scientists and counted 358 sharks (115 h effort), averaging 3.11 sharks per hour. Grey Reef Sharks (*Carcharhinus amblyrhynchos*) comprised 64% of sightings. Nine speared fish were fully depredated by sharks as spearfishers attempted to retrieve their catch, which equates to a depredation rate of 5.9%. The depredated fish included four pelagic fish and five reef fish. The shark species responsible were Grey Reef Shark (*C. amblyrhynchos*) (66%), Bull Shark (*Carcharhinus leucas*) (11%), Whitetip Reef Shark (*Triaenodon obesus*) (11%) and Great Hammerhead (*Sphyrna mokarran*) (11%). There were spatial differences in fish catch, shark sightings and rates of depredation. We developed a report card that compared average catch of fish, sightings of sharks per hour and depredation rate by survey area, which assists recreational fishers and marine park managers to assess spatio-temporal changes. The participating spearfishers can be regarded as experienced (average 18 days a year for average 13.4 years). Sixty percent of interviewees perceived that shark numbers have increased in the past 10 years, 33% indicated no change and 7% indicated shark numbers had decreased. Total fuel use of all vessels was 2819 L and was equivalent to 6.48 tons of greenhouse gas emissions for the competition.

## 1. Introduction

Spearfishing is a highly selective recreational activity that is increasingly popular [1,2,3]. Like other forms of fishing, spearfishing can have rapid and substantial negative effects on reef fish populations [4,5,6,7]. Despite the relative social and economic importance of spearfishing, catch and effort are difficult to monitor and quantify, and its species-specific impacts on the Great Barrier Reef (GBR), and elsewhere, are poorly understood [1,8,9]. In the past 20 years, advances in equipment such as wetsuits, spear guns, masks and boats has undoubtedly increased the potential for spearfishers to spend more time in the water or reach locations farther from shore, and advances in education and sustainability have enabled greater targeting of pelagic species [1,2,9].

A conservation concern is the dramatic global decline in shark numbers [10]. Recent research found that reef sharks were almost completely absent from reefs in several nations, and shark depletion was strongly related to socio-economic conditions such as the size and proximity of markets, inadequate governance and size of the human population [11]. In contrast, high numbers of sharks may be observed in marine protected areas, ecotourism and fishing sites and some protected species have increased in abundance [12].

Shark research has grown in recent decades [13] and data collected by the public can provide a cost-effective means of monitoring populations of wild animals [3,14,15]. Several citizen science initiatives, taking advantage of growing numbers of recreational fishers, Self-Contained Underwater Breathing Apparatus (SCUBA) divers and free divers, have produced information used for conserving and managing fisheries and marine biodiversity [16,17,18]. This information includes data on corals, fish and charismatic fauna such as sharks [19,20,21]. In contrast, the potential benefits of involving spearfishers in research activities have been largely overlooked [22], although this situation is changing [8,23]. Spearfishers are likely to be among the first able to observe, report and respond to potential increases and declines in their target species [9].

In Queensland, Australia, coral reef fish are harvested by the commercial, recreational, Indigenous and charter fishing sectors. The commercial sector is worth around $31 million (2017–2018) at the first point of sale, and there are estimated to be close to 943,000 recreational fishers in Queensland [24]. Approximately 1–2% or 9430–18,860 people are estimated to be spearfishers. Modern spearfishing is a recreational and occasionally competitive activity that occurs from charter vessel, private vessel and shore-based throughout the Great Barrier Reef region, facilitated by clear, warm water, diverse and abundant fish. The most popular target species are *Plectropomus* spp. (Coral trout) [5,24]. Spearfishing activity has restrictions on location, season, protected species, fish sizes (minimum and in some cases maximum length) and equipment (snorkel and not SCUBA, no torches) [25]. Australian spearfishing competitions may involve up to 104 competitors [26,27,28] and have restrictive rules such as (i) capture of only one of each eligible species, (ii) prescribed minimum weights, and (iii) competition during only for a set time (generally five hours).

There have been major changes in spearfishing rules, methods, catch and sustainability [26,27,28], therefore it is timely to generate a better understanding of the scale of spearfishing activity and the potential impacts it has on target species. An example of such change is the increased interest by spearfishers in pelagic fish (such as Dogtooth Tuna, Spanish Mackerel and Wahoo) rather than reef (Groupers, Parrotfish and Sweetlip) and coastal species (Golden Snapper, Barramundi and Stripey) [9,29] over recent years.

Shark depredation, where a shark partially or completely consumes an animal caught by fishing gear before it can be retrieved, occurs in commercial and recreational fisheries worldwide, causing a range of negative biological and economic impacts [30,31]. Shark depredation is a growing source of human-wildlife conflict within the Queensland fishing community. Anecdotal reports suggest that shark depredation has increased over the last 10–20 years. To date, shark depredation has only been quantified in line fisheries in Australia [30,32,33] with no scientific data on the occurrence of shark depredation associated with spearfishing. This is an important data gap because the fish species depredated and the shark species responsible may differ from those associated with line fisheries. In addition, spearfishers can provide a unique insight into depredation as they can observe it directly in the water, enabling them to identify both the fish species taken and the sharks responsible, unlike in most cases of line fishing depredation, where the event often occurs at depth out of sight of the fishers. Furthermore, spearfishers can collect video footage of depredation events to display shark behaviour. Understanding the dynamics of depredation during spearfishing is important because it is intrinsically linked to the potential risk of spearfishers being bitten by sharks.

In order to provide key information on spearfishing in the Great Barrier Reef, this study aims to develop and apply methods to quantify catch, effort and depredation during a national spearfishing competition. Additionally, the study aims to quantify the number and species of sharks observed and their behaviour in relation to spearfishers and speared fish. Finally, our research aims to address a priority of the Queensland Government [34] to investigate fishing depredation and identify the key species driving depredation and assess the extent of depredation.

## 2. Materials and Methods

### 2.1. Study Area, Fisheries Management Arrangements and Competition Rules

The Great Barrier Reef Marine Park (GBRMP) is located on the north-eastern coast of Australia, spanning over 2300 km of coast, 14 degrees latitude and extending from 60 km to 250 km from the coastline. The park covers 344,400 km^2^ in area with an average depth of 35 m, and its greatest depth exceeding 2000 m. The GBRMP is a multiple use area with approximately 34% zoned as Marine National Park (green zone) where fishing is prohibited.

**Figure 1:**
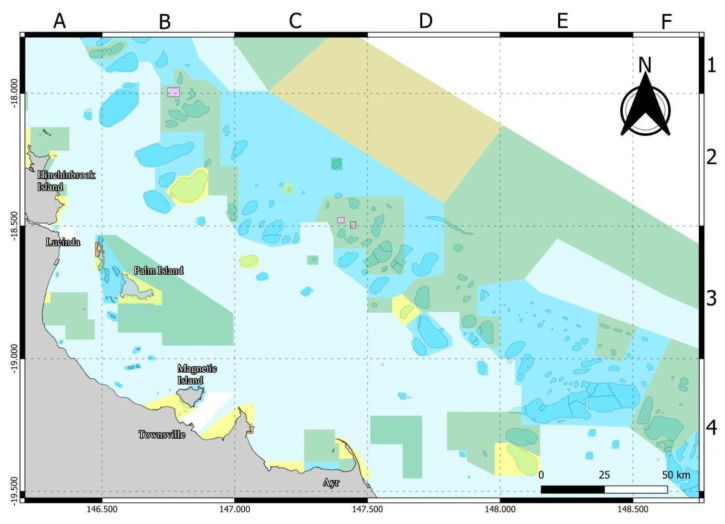
Extent of North Queensland Bluewater Invitational (NQBI) spearfishing competition and the survey location areas within the Great Barrier Reef Marine Park. The blue and yellow zones are waters where spearfishing is permitted and green zones are closed to spearfishing. Grid letters A–F and numbers 1–4 are used to label locations of NQBI competition.

The annual North Queensland Bluewater Invitational (NQBI) spearfishing competition commenced in 2013. We report on the annual event held on 27 and 28 November 2021. The NQBI competitors target pelagic fish species with a prescribed list of “category 1 fish (200 points per fish, plus 10 points per kg (maximum 700 points per fish: all Mackerel (except Shark and Scad), all Tunas, all Billfish, Dolphin fish (also known as Mahi Mahi), Wahoo, all Jobfish, Cobia) and category 2 fish (100 points per fish, plus 10 points per kg (maximum 200 points per fish: Shark Mackerel, all Trevally (except Giant Trevally), all Queenfish, Rainbow Runner, all other pelagic species (except Barracuda) not otherwise detailed)”. The NQBI rules prescribe a maximum of three eligible fish per competitor over two days, one fish per species; and maximum of one (1) category 2 fish per diver. Competitors may also spear other pelagic and reef fish for food or berley. Spearfishing in the NQBI competition is restricted to the area within the GBRMP between Mission Beach (latitude 17°55′ S) south to Cape Upstart (latitude 19°43′ S) (Figure 1). Start time is any time Saturday and finish time is 3pm the following Sunday. All competitors must be members of the Australian Underwater Federation [35].

### 2.2. Questionnaire, Interview and Spearfishing Methods

The design of a survey of shark species and numbers observed and depredation was discussed with Fisheries Queensland and members of the Townsville Skindiving Club prior to the event. Twenty written questions, including location, effort, catch, shark species observed and depredation behaviour, were given to competitors and also asked directly by interview (Appendix A.1). Information on shark species identification, marine park maps (competition area subdivided into 24 grids, Figure 1) and the survey was shared on social media and also included in a folder and waterproof paper provided to competitors.

Spearfishers from each vessel operated as a team. One person remained on-board, and other spearfishers drifted over deep water at the front of reefs or shoals with the assistance of currents. Each spearfisher had a speargun and usually utilised methods to attract pelagic fish, such as a small buoy with a flasher (i.e., a hookless, brightly coloured or reflective, weighted lure) suspended below at 8 to 15 m depth, and/or berley. Spearfishers floated on the surface and occasionally free dived to depths between 2 and 25 m. At the conclusion of a drift (generally over a distance 200–500 m and duration of 30–60 min) the spearfishers reboarded the vessel and moved to another location. Up to 15 drifts and 100 free dives were completed by individual spearfishers during a 10-h day.

The Great White Shark (*Carcharodon carcharias*), Tiger Shark (*Galeocerdo cuvier*) and Bull Shark (*Carcharhinus leucas*) are regarded as potentially dangerous species to humans by the Queensland Government [34]. Visual counts were made by a spearfisher of all sharks sighted in a competition day held between 27 and 28 November 2021. One spearfisher from each vessel recorded the location and numbers of sharks sighted within approximately 20 m of the group. Where possible, photographs and/or videos were obtained to assist with species/group identification of sharks.

Information was recorded on shark species and numbers, date, time period, location, water temperature, water visibility and depth of the seabed where sharks were observed. Additional information on the length and sex of the shark was recorded where possible. Some species that were difficult to identify were grouped to family. A data verification process was established where one of the authors checked for possible errors, viewed photographs and videos and interviewed spearfishers from each vessel.

Species and weight (to 0.01 kg) of eligible pelagic fish were recorded at the completion of the competition. The total number of fish was recorded but species of other fish caught during the competition were not recorded unless depredated by sharks. Catch per unit effort of total fish by vessel was calculated for number of fish and weight of fish.

### 2.3. Statistical Analysis

Data were analysed using R version 4.2.1 [36] and the Primer v7 multivariate program [37] (Table 1, Table S1). Shark sightings were standardised as shark per unit effort (SPUE) while fish catch as catch per unit effort (CPUE), where effort is time spent in hours. Depredation rate was calculated as the percentage of depredated fish from the total number of fish caught and those lost due to depredation. We tested the assumptions of normal distribution using a Shapiro-Wilk test and homogeneity of variance was tested using Levene’s test. Analysis of Variance (ANOVA) and two-sample *t*-tests were used for parametric pairwise comparison and Kruskal-Wallis and Mann-Whitney U tests for non-parametric pairwise comparison. Regression analysis was used to determine the relationship between (1) CPUE and depth, (2) CPUE and SPUE, (3) SPUE and depth, (4) depredation rate and SPUE. Finally, the sighting of shark species was compared as the “assemblage” of sharks among location grids using the permutational multivariate analysis of variance (PERMANOVA) to determine if different locations had different grouping of sharks as observed by spearfishers. The relationship of this assemblage among locations was depicted using non-metric Multidimensional Scaling (nMDS). Both the PERMANOVA and nMDS were based on the Bray-Curtis similarity measure, a standard approach often used to discriminate between locations based on the relative abundance of taxa [37]. For statistically significant results, we performed a *post hoc* Tukey’s honestly significant difference (HSD) test. We recognise that the statistical power of the spatial data was limited due to the small number of observations in each location. Besides the small number of observations in each location, the statistical power of the analysis in this study may also be affected the uneven number of observations between groups in parametric/non-parametric pairwise comparisons.

### 2.4. Assessment to Inform Future Management

We utilized a report card based on the existing framework of the Great Barrier Reef Marine Park Authority Outlook Report [38] to assess the status of three criteria: fish catch, shark sightings and shark depredation associated with spearfishing using a four-point grading scale (Table 2). The status on fish catch, shark sightings and depredation has been collected and summarised in a way that agencies, recreational fishers and others interested in fish and sharks can easily understand and to inform the management of marine parks [38]. Our assessment was based on total data and the three criteria by grid locations in Figure 1.

## 3. Results

### 3.1. Fishing Effort

Nine vessels between 5.5 m and 8 m length, containing 33 competitors, registered for the NQBI competition on the weekend of 27–28 November 2021. A total of 115 h was expended in the water by nine teams (or 268 h based on individual spearfishers) with the majority of competitors commencing around 7 a.m. and finishing around 5 p.m. on day 1, and 7 a.m. and 12 p.m. on day 2. Two daily surveys (Saturday, Sunday) were received from six teams and one daily survey (Saturday) was received from three teams, totalling 15 surveys. The NQBI is a two-day competition and the majority of competitors chose to stay overnight in vessels to reduce travel time and maximise spearfishing time. The daily effort of an individual team of divers in one vessel ranged from 4 to 11.5 h per day during the competition. The average daily time that vessels containing spearfishers searched for fish on day 1 was 9.4 h and the average time for day 2 was 5 h.

**Figure 2:**
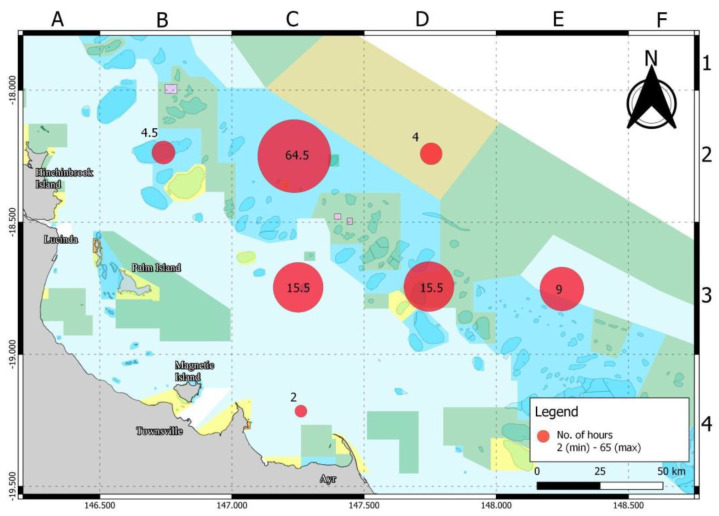
Total effort (hours) of spearfishers between 27–28 November 2021. Grid letters A–F and numbers 1–4 are used to label locations of NQBI competition.

Competitors fished within the boundary of approximately 120 km of coastline offshore from Lucinda and Townsville, with the map indicating areas of highest effort (Figure 2). The effort is spread over multiple locations, reefs and shoals. Spearfishing effort was contained within seven of the possible 24 grids, with the majority of effort located within the grids to the east of Hinchinbrook and Palm islands (Figure 2). Spearfishers reported that 86.6% of vessels had the engine running whilst spearfishing, 100% of spearfishing teams used flashers and 93.3% of teams used berley. Average water depth of spearfishing activity on reefs and shoals ranged between 15 and 40 m, with a reported maximum of 70 m water depth and an average of 25 m. The nine vessels reported that they travelled between 130 and 250 km each day (average 167.3 km) and used between 140 and 300 L of fuel each day (average 187.9l).

### 3.2. Catch

The total catch by spearfishers was 144 fish, including eligible competition fish, reef fish and species captured for berley. Forty-seven eligible fish (or 32.6% of total catch) comprising nine species were presented for weigh-in at the conclusion of the competition (Table 3). The minimum weight of individual eligible fish ranged from 1.75 kg to a maximum weight of 34.4 kg. The total weight of eligible fish was 331.65 kg and the average weight of individual fish was 7.05 kg (Table 2). The average catch per hour of eligible fish by teams was 0.41 fish and 2.88 kg per hour and the average catch by individuals was 0.17 fish and 1.23 kg per hour. Several eligible species including Wahoo (*Acanthocybium solandri*), Mahi Mahi (*Coryphaena hippurus*) and Cobia (*Rachycentron canadum)* were not captured during the competition.

**Figure 3:**
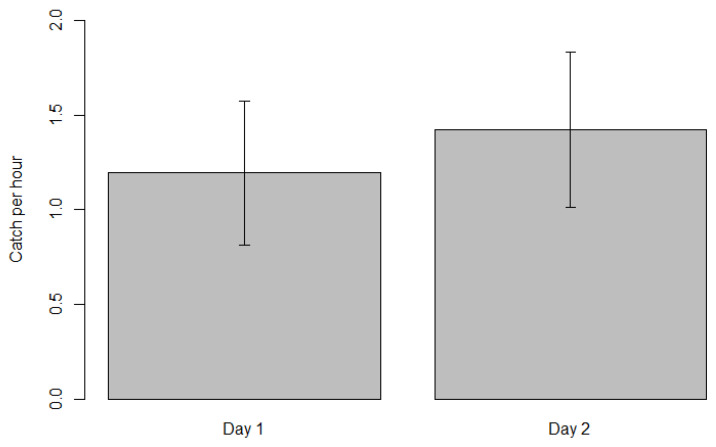

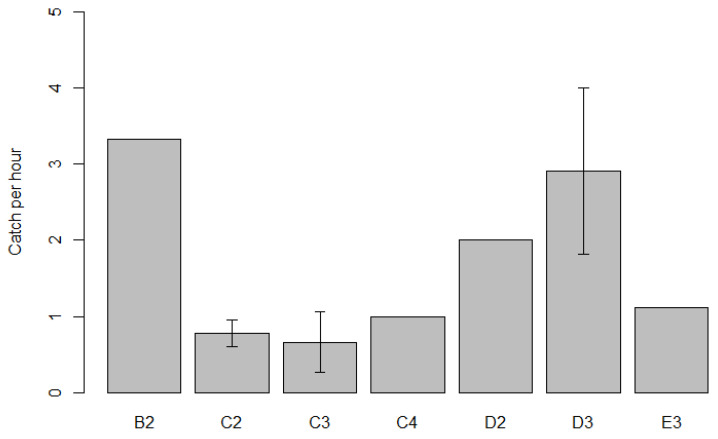
Differences in catch per unit effort (CPUE) ± SE of fish by day (**top**) and by location (**bottom**). See Figure 1 for grid cell locations.

Total average CPUE was 1.25 fish per hour and 0.41 for eligible competition fish per hour. Average CPUE in day 1 and day 2 was 1.2 and 1.42 fish per hour respectively, while grid cells B2 and D3 (Figure 2) recorded the highest CPUE among six locations (Figure 3). Statistical analysis of catch per unit effort (CPUE) did not show significant difference between days and among locations. Regression analysis of CPUE with SPUE (R2 = 0.07, F(_1,14_) = 1.023, *p* = 0.33) and depth (R2 = 0.09, F(_1,14_) = 1.38, *p* = 0.26) did not show any significant correlation.

### 3.3. Shark Sightings

In total, 358 sharks from 10 species/groups were recorded (Table 2). One shark was unidentified either to species or group. Ten species and 270 sharks (or 75%) and five species and 88 individuals (25%) were recorded on days 1 and 2, respectively (Table 4). On average, 3.02 sharks were sighted by spearfishing teams per hour. Grey Reef Sharks were numerically dominant on both days, comprising 64% of all sightings. Far fewer Grey Reef Sharks were recorded on day 2 than on day 1. Whitetip Reef Sharks and Bull Sharks comprised 16% and 12% of sightings, respectively. Interestingly, five Whale Sharks (*Rhinocodon typus*) which are rarely and sporadically observed on the Great Barrier Reef were sighted.

**Figure 4:**
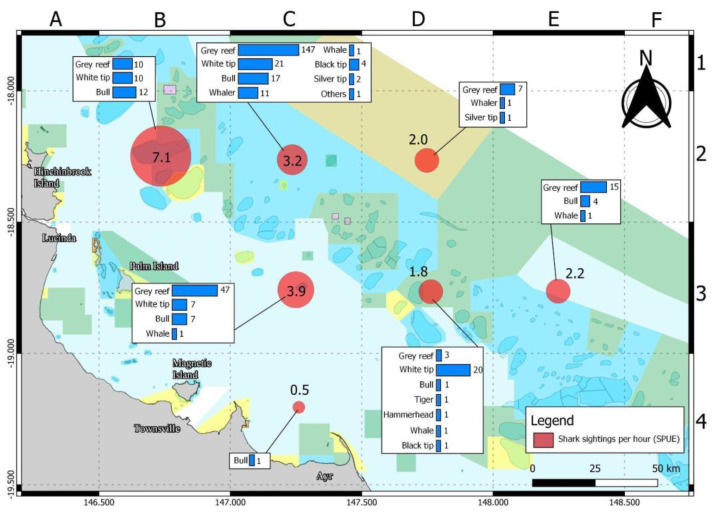
Comparison of shark sightings (per hour) and shark species by survey location on 27–28 November 2021. Grid letters A–F and numbers 1–4 are used to label locations of NQBI competition.

Figure 4 shows the number of species recorded on each site with the highest recorded number in C2 and C3. Highest SPUE was recorded at B2 with observations of 7.1 sharks per hour (Figure 4).

**Figure 5:**
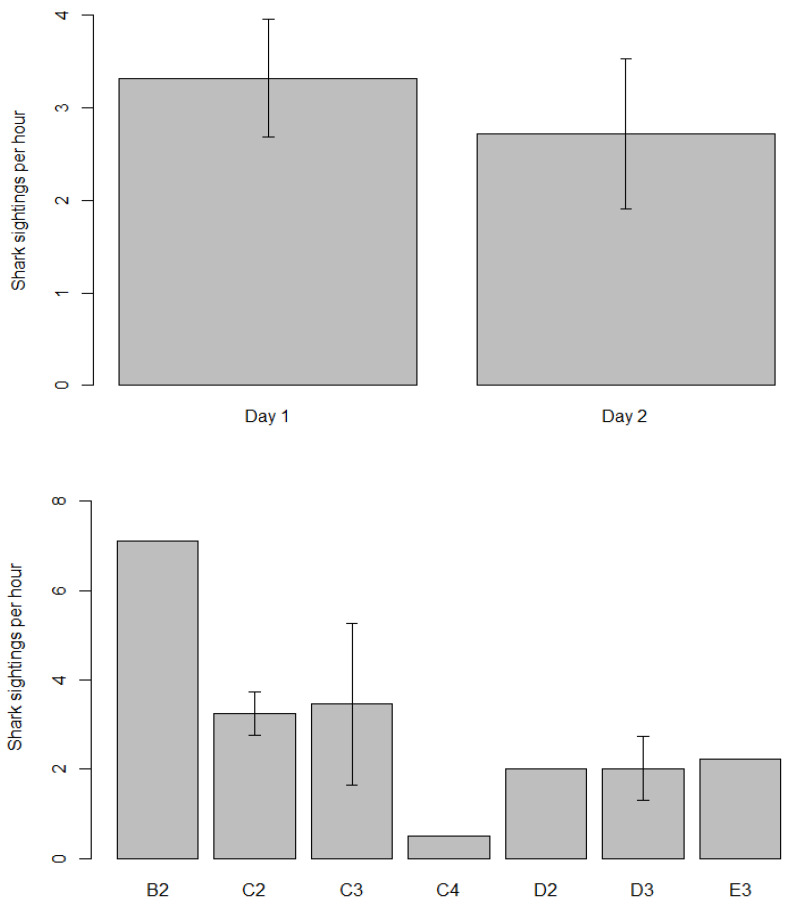
Differences in shark sightings-per-unit-effort (SPUE) ± SE by day (**top**) and by location (**bottom**). See Figure 1 for grid cell locations.

Spearfishers recorded a greater SPUE on Day 1 at 3.3 than on Day 2 at 2.7. The highest SPUE was observed in B2 at 7.1 while C4 with 0.5 had the least (Figure 5). SPUE did not differ significantly between days or among locations. Regression analysis of SPUE and depth show no significant relationship between the two (R2 = 0.03, F(_1,14_) = 0.47, *p* = 0.51).

Statistical analysis of SPUE for each shark species recorded per day showed no significant differences while shark species differed significantly by location for two species (i.e., Whitetip Reef Shark and Bull Shark). A *post hoc* Tukey HSD test for Whitetip Reef Shark (F_(6,9)_ = 4.63, *p* = 0.02) showed significant differences in abundance between C2 and B2. A *post hoc* Tukey HSD test for Bull Shark (F_(6,9)_ = 6.04, *p* = 0.009) showed that abundance differed significantly between B2 and all six other locations.

**Figure 6:**
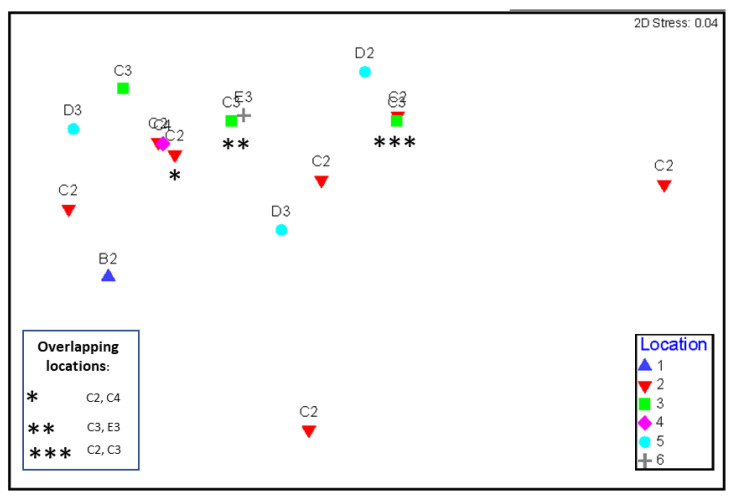
Non-metric multidimensional scaling (NMDS) comparing sightings-per-unit-effort (SPUE) of shark sightings (total assemblage) among locations (see Figure 1 for grid cell locations).

Multivariate analysis comparing the assemblage of shark species likely to depredate showed no significant difference among locations (*p* > 0.9), but there was large variation among sightings by the competing vessels when they visited the same locations (Figure 6). This large variation may be due to other factors, such as spatial variation in sharks within locations (particularly C2), difference in attraction of sharks to vessels or variation in spearfisher methods.

Dangerous sharks [34] comprised 12% of all sharks sighted with one *G. cuvier* and forty two *C. leucas* were reported, and no *C. carcharias* were recorded in our study.

### 3.4. Shark Depredation

**Figure 7:**
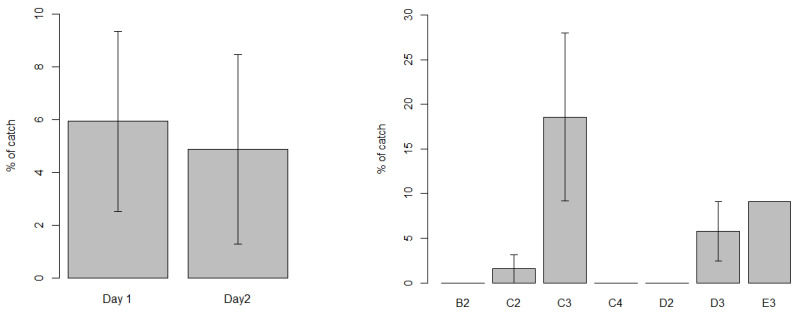
Differences in depredation rates (% of catch) by day (**left**) and location (**right**). See Figure 1 for grid cell locations.

Nine fish (5.9% of the total 153 fish speared) were depredated by sharks. Grey Reef Sharks were the most likely species to depredate fish from spearfishers (Table 5: 66% of all depredations recorded), taking both pelagic (50%) and reef species (50%). Bull, Whitetip and Great Hammerhead sharks each depredated one fish from spearfishers. Single factor ANOVA and two-sample *t*-tests showed no significant differences in shark depredation rates between days (*p* = 0.62) and across locations (*p* = 0.29) (Figure 7). Regression analysis did not show any significant correlation between SPUE and depredation (R2 = 0.11, F(1,14) = 1.76, *p* = 0.21).

### 3.5. Summary of Fish Catch, Shark Sightings and Depredation

**Figure 8:**
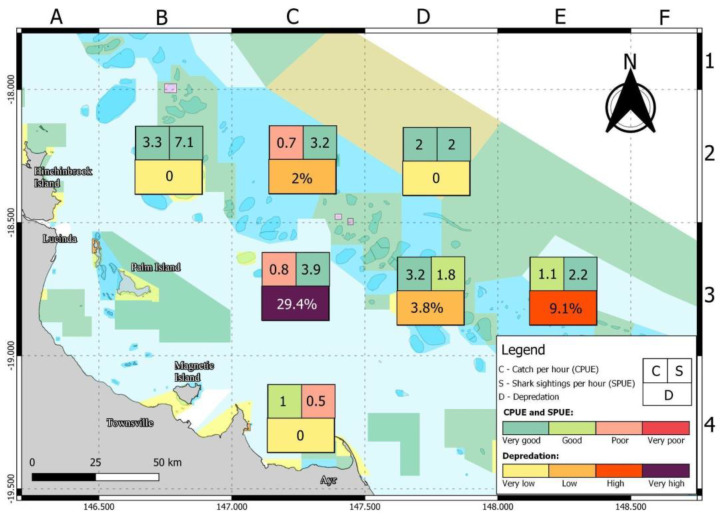
Average catch of fish and sightings of sharks per hour by survey unit area and assessment of status of fish catch, shark numbers and depredation (see Table 2 for key). Grid letters A–F and numbers 1–4 are used to label locations of NQBI competition.

Spatial differences in fish catch, shark sightings and depredation rates are shown in Figure 8. Fish were captured in seven survey areas; sharks were sighted in all seven areas and depredation occurred in four of the areas. The highest rates for fish catch and shark sightings were from a single visit to location B2 (Figure 8). The smallest rate for fish catch was at C2 and C3 (Figure 8). The smallest rate for shark sightings was C4. No fish depredation occurred at B2, C4 or D2 (each also visited only once). The highest depredation rate was at location C3 followed by E3 and D3 (Figure 8), which were all at the same approximate latitude and may be described as mid-shelf reefs.

## 4. Discussion

Marine recreational spearfishers compete for resources with other stakeholders, particularly commercial and recreational anglers and sharks [3,34]. Spearfishers can be highly selective (both in terms of species and fish size) and have specific motivations related to being in touch with the sea, which opens several opportunities for participatory research and management [3,39].

This baseline study is the first scientific study of spearfishing catch, shark diversity and numbers and shark depredation of speared fish. The study identifies the key shark species responsible for depredation of speared fish on the Great Barrier Reef, and assesses the extent of depredation, covering a large area of over 150 km^2^, including the nearshore, reef and shoals. The study did not, however, address the factors influencing depredation.

### 4.1. Spearfishing Effort and Catch

As a result of calm seas and suitable conditions for offshore boating, competitors travelled long distances to the outer Great Barrier Reef shoals and reefs and sought to catch pelagic fish species such as Dogtooth Tuna (*Gymnosarda unicolor*) and Spanish Mackerel (*Scomberomorus commerson*).

A large proportion of spearfishing effort was in one zone (C2) (Figure 2) with lower amounts in six other zones. Three of the zones, E3, B2 and C4 (Figure 2), were visited by spearfishers on one occasion. The unequal effort between zones makes statistical comparison difficult.

Only 32% of the total catch comprised eligible competition species. Most of the catch was “other” pelagic fish and reef species captured for food and/or berley. The average catch of eligible fish per hour by teams was 0.41 fish and 2.88 kg per hour and the average catch by individuals was 0.17 fish and 1.23 kg per hour. This catch is more than the average catch per spearfisher of 0.654 kg/h in the Canary Islands [40] and similar to 1.366 kg/h from the Mediterranean [41].

The spearfishing effort and catch of the North Queensland Bluewater Invitation was similar to other Australian and international competitions [42,43]. Italian spearfishing competitions between 2009–2020 comprised between 8–53 competitors for 4–5 h events and captured 33 species with a mean individual mass of 0.64 ± 0.01 kg and overall CPUE for all the tournaments of 0.47 ± 0.01 kg/spearfisher/h [42]. Our study comprised 33 competitors for an approximately 15 h event (over two days) and captured 8 species of eligible fish with a mean individual mass of 7.05 kg The spearfishing effort and catch were small compared with commercial and recreational line fisheries. The annual effort for the East Coast Spanish Mackerel line fishery for 2018/19 included 164 licenses over 4013 days with CPUE of 66.7 kg/day [44]. In comparison, the daily CPUE for Spanish Mackerel in the NQBI was 8.52 kg/day per vessel.

### 4.2. Shark Observations

Previous research has demonstrated the capability of fishers and citizen scientists in identifying shark species [16,45]. Anglers correctly identified 97.2% of all shark catches submitted during the Texas Shark Rodeo from 2014–2018, although identification would be expected to be higher given that sharks were landed and could be viewed close-up, compared with free-swimming sharks observed when spearfishing. Spearfishers were highly experienced in shark identification and identified 99.72% of sharks. We also requested spearfishers to photograph or video sharks to aid identification.

Typically, scientific underwater visual surveys involve fish and sharks being counted by scientists using SCUBA within delineated boundaries (e.g., belt-transect or stationary point count [46,47,48]). Whereas recreational spearfishers move around a dive site, visually scanning the water column and often moving towards objects of interest. During a previous shark survey, Smith et al. [49] reported that 443 sharks from five species were recorded by spearfishers in the Coral Sea from 30.5 h of freediving (average 14.5 sharks/h), with the most common species being *Carcharhinus amblyrhynchos* (69%). The numerically dominant shark species in the current study was also *C. amblyrhynchos*. Smith et al. [49] reported that 180 sharks were opportunistically recorded by spearfishers in the Great Barrier Reef and the most common species was *Triaenodon obesus* (32%). Sixteen percent of sightings were *T. obesus* in the current study. The possible explanation is that most spearfishing activity in the NQBI was undertaken predominantly in deeper water (average 26 m) rather than shallow water (5–10 m). Rizzari et al. [50] found that *C. amblyrhynchos* prefer deeper sections of reef slope, whereas *T. obesus* are more common in shallower reef areas such as back reef and reef flat [51,52,53].

There are several challenges for surveying sharks and comparing research over time including different locations, depths, times and units: hour (Smith et al. [49], this study) or area (Ayling and Choat [48]). The first difference is SCUBA compared to freediving, because sharks are known to be more wary of SCUBA divers due to the disturbance created by the bubbles [53]. Ayling and Choat [48] surveyed 0–20 m depth (average 10 m) and this study was 5–70 m depth, with an average of 25 m. Spearfishers surveyed locations at the fore reef or pressure point associated with schools of baitfish (Fusiliers, Rainbow Runners and Unicornfish) and Ayling and Choat [48] surveyed fore and back reef areas. In addition, spearfishers in this study used flashers and berley which attracted sharks.

Anecdotally, there appears to be a degree of habituation with sharks attracted to spearfishing activity at more regularly visited locations of C2 and C3 (Figure 2). This may also account for perceptions of greater shark numbers observed in recent times. High densities of 2.94 and 5.5 sharks per hectare (converted to 3.9–7.3 sharks per hour) were reported by Ayling and Choat [48]. Due to roving spearfishers being able to spend more hours in the water per day than SCUBA diving scientists and cover larger areas than most scientific methods, the chance of detecting rare fish and sharks such as *Rhincodon typus* is greater.

Shark abundance has also been measured in this section of the GBR using Baited Remote Underwater Video (BRUV) surveys. These surveys found a total abundance of 2.14 ± 0.27 per hectare for all reef shark species combined in reef slope areas and 1.42 ± 0.23 per hectare in back reef areas [51,52]. Results from these BRUV surveys may be more comparable to the results reported in the current study because an attractant (bait) was used, similar to the berley and flashers used by spearfishers, as opposed to no bait in the research by Ayling and Choat [48]. These results indicate the large differences, challenges and perceptions between numbers of sharks reported by scientists and recreational fishers.

### 4.3. Shark Depredation

Depredation is similar to natural feeding behaviour where sharks opportunistically prey on injured or unhealthy fish, because a speared fish is injured and restricted in its ability to escape. When locating and depredating speared fish, it is likely that the highly evolved sensory systems of sharks respond to a number of biophysical and/or environmental cues. For example, a shark may detect the auditory cues of a boat engine, visual cues of a flasher and odour cues of berley. The sound/vibration of fish struggling after being speared has been shown to attract *Carcharhinus falciformis* and *T. obesus* [54]. There are anecdotal reports of sharks being habituated to some fishing locations (shoals, shipwrecks, islands, bays) and spearfishers often report seeing the same shark on multiple locations [49] which indicate that some sharks are attracted to spearfishing activity.

Shark depredation is not evenly distributed across space and time [30,31]. Mitchell et al. [30] reported substantial spatial variation in depredation rates, with higher depredation in areas that received greater fishing pressure. Our study found that location C3 (Figure 8) had the highest depredation rate by sharks at 29.4%. A possible explanation is that the C3 is the closest location to a large town and has the highest apparent fishing pressure. Carmody et al. [33] reported a positive relationship between depredation rates and proximity to human population centres. Mitchell et al. [30] found that sharks arrived more quickly at fished areas than to no-take zones, and Lester et al. [55] reported a faster time of arrival closer to boat ramps. However, this is counter-intuitive to the general observation of spearfishers that there are more sharks as you travel further offshore (i.e., Coral Sea reefs) and you lose more fish to sharks if you are targeting large pelagic fish rather than reef fish.

Of the 906 fishers interviewed by Ryan et al. [32], 52% indicated they had experienced at least one shark encounter while fishing during the previous year. In contrast, of the 33 spearfishers interviewed in this study, 100% experienced at least one shark encounter each day, with an average of 3.02 shark sightings per hour. However, only nine of the 358 sharks sighted, or 2.5%, successfully depredated caught fish. We note that in some instances multiple sharks depredated a speared fish. Mitchell et al. [30] reported quantitative rates of depredation between 0.9% and 26% in commercial and recreational fisheries worldwide, so the mean rate of 5.9% recorded in the current study is at the lower end of this, however the rate of 29.4% in one of the survey grids was high. Additionally, Mitchell et al. [30] recorded mean depredation rates of 7.2–13.7% in a boat-based recreational fishery in northwest Western Australia and Carmody et al. [33] reported depredation rates of between 1.7–5.7% in a commercial trolling fishery for mackerel across Western Australia. A depredation rate of 8.4% was recorded in a charter fishery operating on the Protea Banks in KwaZulu-Natal, South Africa [56]. The low mean rate of depredation in the current study, compared to the other studies in boat-based fisheries, may have resulted because spearfishers are more selective over the fish they catch and would choose not to spear a fish if there was a shark nearby, whereas fishers on a boat do not know what fish will take the bait or how close sharks are. Likewise, due to the safety risk when multiple sharks are present, spearfishers may choose to leave an area if there are many sharks around, moving to a site where there are fewer sharks and thus a lower risk of depredation occurring.

The depredation ratio of pelagic to reef species in our study was close to 1:1, with 44% pelagic fish and 56% reef fish. However, in the South African study, the depredation rate was recorded to be 18.6% when targeting pelagic species and 1.9% when targeting reef species, which represents a 10:1 ratio [56]. Mitchell et al. [30] reported a mean depredation rate of 11.5–13.7% for demersal fishing and 7.2–11.8% for trolling for pelagic species.

All of the shark species that depredated fish during the NQBI were identified to species. *Carcharhinus amblyrhynchos* was the most commonly observed shark species and appeared to be most abundant in water depths of 25–40 m (pers. obs.). Sixty-six percent of the shark depredation events in this study were attributed to *C. amblyrhynchos* and eleven percent (or one incident) each to Bull Sharks, Great Hammerheads and Whitetip Reef Sharks. Vardon et al. [57] identified shark species responsible for fisheries depredation off Southeast Queensland using mitochondrial DNA collected from swabs of depredated fish. All depredating shark species identified were in the genus *Carcharhinus* and greatest depredation was recorded by Bull Sharks (*n* = 5).

Sumner et al. [58], Williamson et al. [59] and Coulson et al. [60] reported that depredation rates ranged widely by species in northwest Western Australia, with most species having depredation rates <2%, but certain key species had higher depredation rates, including *Glaucosoma hebraicum*, *Scomberomorus commerson* (6.08% and 13.3%), *Lethrinus nebulosus* (4.7%) and *Plectropomus* spp. (4.6%), which was partly caused by the fact that fishers commonly targeted these species. Our study also found high rates of depredation for *S. commerson* which was the most common pelagic fish weighed in the competition (*n* = 20) and depredation rate of 9.1% was higher than the average of 6.2% for all fish.

A recent study by Hoel et al. [61] used a Levels of Conflict framework to explore the social dimensions of shark depredation in Queensland. This work used semi-structured interviews to collect information from 12 line fishers (from commercial, charter and recreational fishing sectors) about their experiences of depredation [61]. This approach revealed that fishers have experienced an increase in depredation over the past 5–10 years and that the behaviour of sharks has changed, whereby they have learnt strategies to follow fishing vessels and depredate catch [61]. The study also revealed deeper conflicts, such as a distrust between fishers and managers and a mismatch between data presented in scientific studies on shark populations compared to their local experiences [61]. The research emphasised the need to include fisher knowledge and values in any research on shark depredation and develop management tools that incorporate a broader range of social-ecological factors [61].

### 4.4. Measures for Reducing Shark Depredation

Measures by spearfishers in response to shark depredation could have management and economic implications, varying from re-distributions of fishing effort (including location and target species) to uptake of expensive shark mitigation equipment (e.g., Shark Shield), or retaliatory killing of sharks.

In order to develop future strategies for reducing depredation, it is necessary to identify the spearfisher effort and techniques, the shark species responsible for depredation and the shark behavioural processes involved. Also, it is important to generate a greater understanding of how depredation rates vary in space and time in relation to environmental changes. This study identified that one of the areas fished (C3; Figure 8) had significantly higher depredation by sharks than other areas during the NQBI, which may be linked to the level of fishing activity that occurs at this location, the abundance of sharks and habitat/environmental factors.

The process of spearfishing is characterized not only by fitness and skill of the spearfisher, gear type, location and water visibility, but also by the opportunities, constraints and decisions that fishers face–such as proximity to number and type of sharks [62]. Spearfishers in the NQBI, Australia make decisions on which fish to target based on competition points (category 1 fish are twice as valuable as category 2), weight (larger fish are worth more points), proximity to the fish (being closer to the fish increases the opportunity for a ‘stone’ shot that kills the fish instantly), proximity to large sharks (it is poor judgment to spear a fish if it is high risk to be depredated by a shark) and proximity to teammates (who can assist in landing the fish and discouraging sharks).

The behaviour of SCUBA divers and sharks was studied by Cubero-Pardo et al. [54] and they considered four discrete categories of shark reaction (evasion, spontaneous approach, alert and no reaction) and five categories of diver behaviour (direct approach, camera flash, sudden movement, noise and simple presence), two categories of observation strategy (still and movement) and the distance of the focal diver group to the sharks. There is no similar study of shark and spearfisher behaviour and no scientifically proven technical or non-technical measure for preventing or reducing shark depredation. Spearfishers in the NQBI used large, multi-rubber ‘bluewater’ spearguns to increase the chance of a successful fish catch in a short time (generally between 30 and 120 s). No spearfishers in the NQBI reported using technical shark mitigation equipment such as Shark Shields, although these are becoming increasingly popular as human risk mitigation from shark bites. All spearfishers in the NQBI adopted non-technical measures such as teamwork and nearby support vessels to remove fish rapidly from the water for prevention or mitigation of shark depredation.

### 4.5. Sustainability

Spearfishing can be considered as a complex, adaptive social-ecological system characterized by behavioural interactions and other feedback mechanisms between humans, fish and ecosystems [3,62]. Spearfishing allows fishers to target the size and species of his\her capture without the negative impacts of other fishing methods such as bycatch, bait, loss of gear and damage to habitat [29]. Spearfishing competitions or tournaments have the potential to concentrate fishing effort in a particular area at a particular point in time. In recognition of this sustainability issue, many competitions (including the NQBI) introduce rules beyond that of marine parks and fisheries agencies. For example, the NQBI allows a maximum of three eligible fish per competitor to be weighed compared to the daily bag limit of 20 reef species.

The sustainability of fishing can be measured in many ways including fish stocks, environmental impact and effective management [29,63]. A relatively new consideration is the environmental impact caused by greenhouse gas emissions associated with travel. Spearfishers traveled long distances and consumed considerable vessel fuel to catch fish. An average of 19.6 L of vessel fuel was consumed for each landed fish. At an estimated cost of AUS $1.60 per litre this is equivalent to $31.32 for each fish. Climate change is a global issue and actions such as reducing or offsetting greenhouse gas emissions must be considered by all citizens. The total fuel consumed by vessels during the NQBI was 2819 L. Burning 1 L of gasoline produces approximately 2.3 kg of CO_2_, which equates to a total of 6.48 tons for all competitors for the competition. In comparison the greenhouse gas emission of 90 tourism operators on the Great Barrier Reef during 2010–2011 was 5,013,421 tons [64]. There is no regulatory or voluntary process to easily offset emissions from recreational fishing vessels or competitions. By quantifying the impact, we can discuss and act for improving the sustainability of future competitions.

## 5. Conclusions

Spearfishers captured 144 fish at a rate of 0.41 fish and 2.88 kg per hour and reported sightings of 358 sharks with an average of 3.02 sharks per hour. The relatively high number of shark sightings may be explained by deep water, proximity of baitfish, the use of flashers, berley and spearfishing. Nine (5.9%) speared fish were fully depredated by sharks, predominantly *Carcharhinus amblyrhynchos*.

Managing conflict over competition between sharks and humans is complex as it has multiple political, environmental, social, economic and legal dimensions affecting a range of stakeholders. The majority (60%) of spearfishers perceived that shark numbers had increased in the past 10 years. However, this generalisation may be problematic because there are estimated 133 species of sharks inhabiting waters of the Great Barrier Reef and some species may have increased and some may have decreased over different periods of time. It is suggested that future research asks questions on specific shark species; for example, “Do you think Bull Shark numbers have increased or decreased in the past 10 years?“ Citizen science research by spearfishers can be useful for fisheries, marine park management and sustainability.

Citizen science participation and communication are vital in sharing knowledge with stakeholders about the status of fish, sharks and shark depredation. We recommend that this citizen science research and reporting framework continue annually for the NQBI spearfishing competition and be extended to other spearfishing and line fishing events in Australia and around the world. We have adopted the methodology and gradings from the Great Barrier Reef Marine Park (GBRMP) Outlook report [38] to summarise the status of fish catch and shark sightings and the impacts of shark depredation, to provide a simple tool that can be communicated and understood by spearfishers and fishers. This knowledge could be integrated into future Outlook reports (every five years) to compare changes over time and to inform future management of the marine park.

## Figures and Tables

**Table 1:** Statistical analysis performed to compare different variables. SPUE (shark per unit effort), ANOVA (Analysis of Variance), CPUE (catch per unit effort), PERMANOVA (permutational multivariate analysis of variance), nMDS (non-metric Multidimensional Scaling).

Questions	Independent Variable	Dependent Variable	Statistical Test
1. Were there any differences in shark sightings in different location grids and competition days?	Time (competition day)	SPUE (number of sharks observed per hour)	Student’s *t*-test
	Location (refer to Figure 1)		Analysis of Variance (ANOVA)
2. Were there any differences in catch per unit effort in different location grids and competition days?	Time (competition day)	CPUE (fish catch per hour)	Mann-Whitney test
	Location (refer to Figure 1)		Kruskal-Wallis
3. What is the most common shark species observed?	Shark species	SPUE (number of sharks observed per hour)	Kruskal-Wallis
4. What is the relationship between fish catch, depth (bathymetry), and shark sightings?	Depth	SPUE (number of sharks observed per hour)	Regression analysis
	SPUE (number of sharks observed per hour)	CPUE (fish catch per hour)	
5. What is the relationship between depredation rate and shark sightings?	SPUE (number of sharks observed per hour)	Depredation rate	Regression analysis
6. Were there any differences in depredation rate in different location grid and competition days?	Time (competition day)	Depredation rate	Mann-Whitney test
	Location (refer to Figure 1)		Kruskal-Wallis
7. Were there any differences in the assemblage of shark sightings among location grids?	Location (days pooled)	SPUE	PERMANOVA and nMDS

**Table 2:** Indicators for assessing status of fish catch (per hour) and shark sightings (per hour) and impact of shark depredation (categories based on grading categories from GBRMPA 2019).

**Status**	**Very Good**	**Good**	**Poor**	**Very Poor**
Fish catch (per hour)	2+	1–2	0.5–0.99	0–0.49
Shark sightings (per hour)	2+	1–2	0.5–0.99	0–0.49
**Impact**	**Very Low**	**Low**	**High**	**Very High**
Depredation (%)	0–1	1–5	5.01–15	>15

**Table 3:** Species, number and weight of eligible fish caught by competitors during the North Queensland Bluewater International (NQBI) in 2021.

Common Name	Scientific Name	Number	Weight (kg)		
			Min	Max	Average
Spanish Mackerel	*Scomberomorus commerson*	20	4.3	11.6	6.8
Grey Mackerel	*Scomberomorus semifasciatus*	1	1.7	1.7	1.7
Shark Mackerel	*Grammatorcynus bicarinatus*	2	3.4	4.3	3.9
Dogtooth Tuna	*Gymnosarda unicolor*	7	5.1	20.5	13.7
Green Jobfish	*Aprion virescens*	2	4.5	4.7	4.6
Turrum	*Carangoides fulvoguttatus*	6	5	7.4	6.2
Rainbow Runner	*Elagatis bipinnulata*	2	2.3	2.4	2.35
Queenfish	*Scomberoides commersonnianus*	1	4.1	4.1	4.1
Sailfish	*Istiophorus platypterus*	1	34.4	34.4	34.4

**Table 4:** Comparison of shark sightings recorded by species and day by spearfishers on the Great Barrier Reef during the NQBI competition.

Common Name	Scientific Name	Day 1	Day 2	Total
Whitetip Reef Shark	*Triaenodon obesus*	37	21	58
Grey Reef Shark	*Carcharhinus amblyrhynchos*	185	44	229
Blacktip Reef Shark	*Carcharhinus melanopterus*	4	1	5
Bull Shark	*Carcharhinus leucas*	21	21	42
Silvertip Shark	*Carcharhinus albimarginatus*	4	1	5
Tiger Shark	*Gaelocerdo cuvier*	1	0	1
Silky Shark	*Carcharhinus falciformis*	1	0	1
Great Hammerhead	*Sphyrna mokarran*	1	0	1
Whale Shark	*Rhinocodon typus*	5	0	5
Whaler species	*Carcharhinus* spp.	10	0	10
Unidentified		1	0	1
Total		270	88	358

**Table 5:** Species of fish depredated by sharks during the NQBI in 2021. Key: P—Pelagic, R—Reef.

Common Name	Scientific Name	Type	Number	Depredating Shark
Spanish Mackerel	*Scomberomorus commerson*	P	2	Grey Reef Shark
Rainbow Runner	*Elagatis bipinnulata*	P	1	Grey Reef Shark
Turrum	*Carangoides fulvoguttatus*	P	1	Bull Shark
Coral Trout	*Plectropomus* spp.	R	3	Grey Reef Shark
Coral Trout	*Plectropomus* spp.	R	1	Whitetip Reef Shark
Steephead Parrotfish	*Chlorurus microrhinos*	R	1	Great Hammerhead
Total			9	

## Data Availability

Not applicable.

## Supplementary Materials

The following supporting information can be downloaded at: https://www.mdpi.com/article/10.3390/biology11101524/s1, Table S1: Table version of the statistical test.

## Appendix A

### Appendix A.1. Spearfishers and Sharks Survey 2021

We would greatly appreciate if one diver for each vessel can be responsible for recording important information each day/location. By collecting research we can improve knowledge and safety of divers. Please return forms to Adam Smith at the weigh-in and he will also check and do any follow up interviews.

1. Approximate location (see attached map or name reef, island or shoal)
2. How many divers in water?
3. What time did divers enter the water?
4. What time did divers leave the water?
5. How many other boats nearby?
6. Did boats have engines running during fishing?
7. Was berleying used?
8. Were flashers used?
9. What was the average depth of water?
10. How many fish did you catch, including both those kept and used for berley
11. Did you see any sharks?
12. If yes, what species of shark and estimated sizes did you see?
  **Species**	**Number**	**Estimated size**	**Photo/Video?**
  Grey reef shark			
  White tip reef shark			
  Bull shark			
  Whaler shark			
  Other			
13. Did you experience sharks eating any of your fish (depredation)?
  If yes, how many fish were partly or completely depredated by sharks?

**Species of Fish**	**Estimated Size of Fish (cm)**	**Species of Shark(s)**	**Partial or Complete**
			
			
			

Other questions

14. Have you been interviewed about shark depredation before?
15. How many days have you spearfishing from a boat in the last year?
16. How many years have you been spearfishing for?
17. Do you think shark numbers have increased or decreased in the past 10 years?
18. Approximately how many kilometres did you travel over the competition?
19. Approximately how many litres of fuel did you use of the competition?
20. Any other observations or comments?

Boat name/number?

Boat length?

Skipper name?

Please return form to Adam Smith adam.smith@reefecologic.org 0418726584

All forms go into a draw for a prize
