# Supplementary material for: Quantifying Catch Rates, Shark Abundance and Depredation Rate at a Spearfishing Competition on the Great Barrier Reef, Australia
Source: Biology (Basel). 2022 Oct 18;11(10):1524. doi: 10.3390/biology11101524 (PMC9598298; doi:10.3390/biology11101524)
Supplement: Supplementary file 1 [file biology-11-01524-s001.zip › biology-1903819-Supplementary-Materials.pdf]

Table S1. Table version of the statistical test.

Independent variable	Dependent variable	Statistical test	Check for assumptions
Time (competition day)	SPUE (number of sharks observed per hour)	Student’s t-test	Shapiro-Wilk normality test<br>W = 0.887, p-value = 0.04995<br>Levene's Test for Homogeneity of Variance (center = median)<br>Df = 1, F value = 0.0256 Pr(>F) group = 0.8752
Location (refer to Figure 1)	SPUE (number of sharks observed per hour)		Shapiro-Wilk normality test<br>W = 0.96277, p-value = 0.7123<br>Levene's Test for Homogeneity of Variance (center = median)<br>Df = 6, F value = 0. 0.5085 Pr(>F) group = 0.7883
Time (competition day)	CPUE (fish catch per hour)	Mann-Whitney test	Shapiro-Wilk normality test<br>W = 0.86958, p-value = 0.02679<br>Levene's Test for Homogeneity of Variance (center = median)<br>Df = 1, F value = 0.0759 Pr(>F) group = 0.787<br>Mann-Whitney U-test<br>W = 25, p-value = 0.5251
Location (refer to Figure 1)	CPUE (fish catch per hour)	Kruskal-Wallis	Shapiro-Wilk normality test<br>W = 0.86958, p-value = 0.02679<br>Levene's Test for Homogeneity of Variance (center = median)<br>Df = 6, F value = 3.1744 Pr(>F) group = 0.05864<br>Kruskal-Wallis rank sum test<br>Kruskal-Wallis chi-squared = 8.987, df = 6, p-value = 0.1743
Shark species	SPUE (number of sharks observed per hour)	Kruskal-Wallis	Shapiro-Wilk normality test<br>W = 0.46709, p-value < 2.2e-16<br>Levene's Test for Homogeneity of Variance (center = median)<br>Df = 8, F value = 12.02 Pr(>F) group = 7.335e-13<br>Kruskal-Wallis rank sum test<br>Kruskal-Wallis chi-squared = 64.879, df = 8, p-value = 5.1e-11
Depth	SPUE (number of sharks observed per hour)	Regression analysis	$y = 0.0381x + 2.1291$<br>$R^2 = 0.0323$
SPUE (number of sharks observed per hour)	CPUE (fish catch per hour)	Regression analysis	$y = 0.1437x + 0.8541$<br>$R^2 = 0.0681$
SPUE (number of sharks observed per hour)	Depredation rate	Regression analysis	$y = 0.017x + 0.015$<br>$R^2 = 0.1018$

Time (competition day)	Depredation rate	Mann-Whitney test	Shapiro-Wilk normality test<br>W = 0.64463, p-value = 4.408e-05<br>Levene's Test for Homogeneity of Variance (center = median)<br>Df = 1, F value = 0.0451 Pr(>F) group = 0.8348<br>Mann-Whitney U-test<br>W = 36, p-value = 0.6263
Location (refer to Figure 1)	Depredation rate	Kruskal-Wallis	Shapiro-Wilk normality test<br>W = 0.64463, p-value = 4.408e-05<br>Levene's Test for Homogeneity of Variance (center = median)<br>Df = 6, F value = 0.6961 Pr(>F) group = 0.6601<br>Kruskal-Wallis rank sum test<br>Kruskal-Wallis chi-squared = 7.3996, df = 6, p-value = 0.2855
Location (days pooled)	SPUE (number of sharks observed per hour)	PERMANOVA and nMDS	
